# Weekly work hours and cervical spine dysfunction among video display terminal workers: mediating roles of occupational stress and sleep quality

**DOI:** 10.3389/fpubh.2026.1798795

**Published:** 2026-06-01

**Authors:** Liuzhuo Zhang, Aipin Xiao, Jiao Bai, Mengli Liu, Yu Hu, Dexiang Zhu, Shaofan Weng, Xizhi Wang, Guangtao Yang, Dafeng Lin, Naixing Zhang

**Affiliations:** 1 Shenzhen Prevention and Treatment Center for Occupational Diseases, Shenzhen, China; 2 School of Public Health, Southern Medical University, Guangzhou, China

**Keywords:** cervical spine dysfunction, mediation analysis, occupational stress, sleep quality, video display terminal workers, weekly work hours

## Abstract

**Background:**

Cervical spine dysfunction is common among video display terminal (VDT) workers. Although long work hours have been linked to musculoskeletal problems, how extended work hours relate to cervical spine dysfunction via psychosocial and behavioral factors remains insufficiently characterized. We examined the association between weekly work hours and cervical spine dysfunction and explored the potential mediating roles of occupational stress and sleep quality.

**Methods:**

A cross-sectional study was conducted among 929 VDT workers from 10 companies in Shenzhen, China. Cervical spine dysfunction was assessed using the Neck Disability Index and dichotomized as dysfunction (NDI > 4) versus no dysfunction (NDI ≤ 4). Weekly work hours were categorized as ≤40, 41–48, 49–56, and ≥57 h/week. Occupational stress was measured using the Job Content Questionnaire (JCQ-22) and defined as a demand–control ratio >1. Sleep quality was assessed using the Pittsburgh Sleep Quality Index and categorized as poor (PSQI >5) versus good (PSQI ≤5). Multivariable logistic regression models adjusted for prespecified confounders were used. Mediation analyses were conducted using single- and parallel-mediator models; 5,000 bootstrap resamples were used for single-mediator models.

**Results:**

Longer weekly working hours were associated with higher odds of cervical spine dysfunction (adjusted ORs 2.10–2.70 vs. ≤ 40 h/week; all *p* ≤ 0.005). Occupational stress was associated with higher odds of cervical spine dysfunction (OR 1.62, 95% CI 1.29–2.03, *p* < 0.001), whereas good sleep quality was associated with lower odds (OR 0.25, 95% CI 0.19–0.32, *p* < 0.001). Weekly working hours were also associated with increased occupational stress (all *p* ≤ 0.002) and poorer sleep quality in some categories. In single-mediator models, the indirect effect via occupational stress was significant (*p* = 0.003), whereas the indirect effect via sleep quality was marginal (*p* = 0.068). In the parallel mediation model, indirect effects via occupational stress (IE 0.04, 95% CI 0.01–0.06, *p* = 0.009) and sleep quality (IE 0.06, 95% CI 0.01–0.11, *p* = 0.019) were significant, and the direct effect was not statistically significant (DE 0.05, 95% CI −0.05 to 0.15, *p* = 0.356).

**Conclusion:**

Longer weekly work hours were associated with higher odds of cervical spine dysfunction among VDT workers. This association may be partly explained through indirect pathways involving occupational stress and sleep quality, supporting prevention strategies integrating working-time management with ergonomic/posture interventions, stress reduction, and sleep health promotion.

## Introduction

Video display terminal (VDT) work has become a dominant occupational pattern in modern workplaces, accompanied by an increasing burden of work-related musculoskeletal disorders (WMSDs), particularly cervical spine dysfunction (1–3). Cervical spine dysfunction can lead to persistent pain, functional limitation, reduced work ability, and substantial productivity losses, underscoring its occupational and public health significance (1–3). Studies commonly group WMSD determinants into four broad categories: (1) biomechanical/biodynamic factors (e.g., awkward neck posture, sustained static loading, repetitive movements, and muscle fatigue), (2) psychosocial factors (e.g., occupational stress and mental workload), (3) individual factors (e.g., age, sex, lifestyle behaviors, and personality-related characteristics), and (4) organizational/work-design factors (e.g., long work hours, insufficient recovery, and shift schedules). Evidence across occupational settings supports these categories, including findings linking subjective mental workload to musculoskeletal complaints (4), individual and personality-related characteristics to WMSD risk using path-analysis approaches (5), biodynamic exposures such as repetitive-task cycles to trapezius fatigue (6), and “hidden” psychosocial/organizational risk factors with sleep as a mediator of musculoskeletal discomfort (7). Prolonged work hours, sustained sedentary behavior, and constrained postures have been associated with musculoskeletal symptoms among VDT workers (1–3); however, biomechanical factors alone do not fully explain the elevated risk of cervical spine dysfunction observed in this population (2, 8). In addition, monotonous computer-based tasks and low task variability may contribute to musculoskeletal complaints by increasing perceived stress, boredom, and sustained somatic tension during prolonged human–computer interaction (9). Increasing evidence suggests that psychosocial and behavioral factors, such as occupational stress and sleep quality, are closely linked to musculoskeletal health and may play an important role in the development of cervical spine dysfunction (8, 10, 11).

Despite these findings, existing studies have often examined long work hours, occupational stress, and sleep-related factors in isolation (2, 8, 12), and the pathways through which extended work hours influence cervical spine dysfunction remain insufficiently understood. In particular, the mediating roles of occupational stress and sleep quality have rarely been investigated simultaneously (8, 12, 13). Conceptually, occupational stress was framed using the Karasek demand–control model (operationalized by the Job Content Questionnaire) (14), and the potential links between work exposure, stress, sleep, and cervical dysfunction were interpreted within a biopsychosocial model of musculoskeletal pain and disability (15). Accordingly, we hypothesized that long work hours may be associated with cervical spine dysfunction through psychosocial stress and sleep quality. Therefore, the present study aimed to examine the association between weekly work hours and cervical spine dysfunction among VDT workers, and to explore the potential mediating roles of occupational stress and sleep quality using multivariable regression and mediation analyses.

## Methods

### Study design and participants

A cross-sectional study was conducted among video display terminal (VDT) workers recruited from 10 companies in Shenzhen, China, with the specific aim of examining differences in cervical spine dysfunction according to weekly work-hour categories within a VDT-exposed occupational population. These companies covered a variety of typical VDT-intensive working environments and included multiple departments with diverse job characteristics. Eligible workers were invited to participate during routine occupational health examinations. The inclusion criteria were: (1) individuals who worked with VDT for ≥4 h per workday and with ≥1 year of service, using devices such as keyboards, mice, or touchscreens; and (2) voluntary participation in the survey with signed informed consent. Exclusion criteria included: (1) individuals with severe cervical spine conditions, such as fractures, dislocations, or spinal cord injuries requiring surgical treatment; and (2) pregnant or lactating women.

All participants were informed of the purpose and procedures of the study prior to participation, and written informed consent was obtained. Ethical approval was obtained from the Ethical Review Committee of the Shenzhen Prevention and Treatment Center for Occupational Diseases (LL2020-34), and was conducted in accordance with the Declaration of Helsinki. Clinical trial number: not applicable.

### Assessment of cervical spine dysfunction

Cervical spine dysfunction was assessed using the Neck Disability Index (NDI), a widely used clinical tool for assessing the functional status of the neck in individuals with acute or chronic neck pain (16, 17). Participants were asked to report symptoms including pain intensity, personal care, lifting, reading, headaches, concentration, work, driving, sleep, and leisure activities. Each item is scored on a 5-point scale, with a total score ranging from 0 (no dysfunction) to 50 (total dysfunction). Higher scores indicate greater dysfunction. In this study, NDI scores were classified as follows: 0–4 (no dysfunction), 5–14 (mild dysfunction), 15–24 (moderate dysfunction), 25–35 (severe dysfunction), and >35 (total dysfunction). For analysis, scores ≤4 were categorized as no dysfunction, and scores >4 were categorized as having dysfunction. Participants were categorized into two groups according to the presence or absence of cervical spine dysfunction. The scale demonstrated good reliability, with a Cronbach’s alpha coefficient of 0.861.

### Assessment of weekly work hours

Weekly work hours were self-reported by participants and categorized into four groups according to commonly used occupational health definitions: ≤40 h, 41–48 h, 49–56 h, and ≥57 h per week. These categories were selected to reflect standard work hours and increasing levels of long work-hour exposure (18).

### Assessment of occupational stress

Occupational stress was evaluated using the Job Content Questionnaire (JCQ-22) (14, 19). The stress level was evaluated using the ratio of the score between job demands and job control (D/C × 9/5). A ratio of >1 indicated the presence of occupational stress, and a ratio of ≤1 indicated the absence of occupational stress. The Cronbach’s alpha coefficient for this scale is 0.749.

### Assessment of sleep quality

Sleep quality was assessed using Pittsburgh Sleep Quality Index (PSQI) (20, 21). It involved seven components, with each component scored from 0 to 3, resulting in a total PSQI score ranging from 0 to 21. The total PSQI score was calculated as the sum of these component scores. Higher scores indicated poorer sleep quality. In this study, scores>5 were defined as poor sleep quality, while scores≤5 were considered good sleep quality. The PSQI is a multidimensional instrument designed to capture multiple aspects of sleep. Therefore, internal consistency as measured by Cronbach’s alpha may not fully reflect the reliability of the global PSQI score.

### Assessment of covariates

Based on previous literature, potential confounding variables were identified *a priori* and included age, gender, marital status, education level, smoking status, drinking status, physical exercise status, and length of employment (22, 23). These variables were collected through structured questionnaires and categorized according to standard epidemiological definitions. Covariates were limited to variables available in the survey dataset.

### Statistical analysis

Descriptive statistics were used to summarize the characteristics of the study population. Continuous variables were presented as means with standard deviations, while categorical variables were expressed as numbers and percentages. Differences between participants with and without cervical spine dysfunction were assessed using the chi-square test for categorical variables.

Multivariable logistic regression models were applied to examine the associations of weekly work hours, occupational stress, and sleep quality with cervical spine dysfunction. Odds ratios (ORs) and 95% confidence intervals (CIs) were calculated after adjustment for potential confounders. Variance inflation factors (VIFs) were calculated to assess multicollinearity among independent variables. To account for clustering of participants within companies, we computed cluster-robust standard errors with clustering at the company level, and reported cluster-robust 95% CIs and *p* values for the logistic regression results.

Mediation analyses were conducted in R (version 4.3.1), and a two-sided p value < 0.05 was considered statistically significant. For single-mediator models, we used the R package mediation (function mediate) to estimate the average causal mediation effect (ACME), average direct effect (ADE), and total effect. We obtained 95% confidence intervals using a nonparametric bootstrap with 5,000 resamples (percentile-based intervals). In addition, we conducted a natural effects model analysis using the R package medflex (neImpute/neModel) as a robustness check. For the parallel and serial mediation models with categorical mediators and outcome, we fitted structural equation models using the R package lavaan with the WLSMV estimator; standard errors were computed using the robust estimator under WLSMV, and confidence intervals for indirect effects were obtained using the delta method based on robust standard errors.

To address potential non-monotonic dose–response patterns, we conducted an additional parallel mediation analysis treating weekly work hours as a categorical exposure (dummy-coded with ≤40 h/week as the reference). We further examined nonlinearity using orthogonal polynomial coding (linear and quadratic terms) as a sensitivity analysis. To address potential information loss due to mediator dichotomization, we repeated the parallel mediation model using the JCQ demand–control ratio (JCQ_rate) and the PSQI total score (PSQI_score) as continuous mediators. In this sensitivity analysis, weekly work hours were modeled as an ordinal term. We also tested a serial (chain) mediation model as a sensitivity analysis to evaluate the hypothesized stress–sleep pathway, specifying weekly work hours as the exposure, occupational stress as the first mediator, sleep quality as the second mediator, and cervical spine dysfunction as the outcome.

## Results

### Participant characteristics

A total of 929 video display terminal (VDT) workers were included in the analysis. Of these participants, 369 (39.7%) were classified as having cervical spine dysfunction. The distributions of demographic characteristics, lifestyle factors, and work-related variables according to cervical spine dysfunction status are presented in Table 1. Significant differences were observed between participants with and without cervical spine dysfunction in terms of gender, age, marital status, education level, physical exercise, drinking status, weekly work hours, occupational stress, and sleep quality (all *p* < 0.05).

**Table 1:** Baseline characteristics of study participants *n* (%).

Variable	Total	Cervical spine dysfunction		*P*
		No	Yes	
	929 (100)	560 (60.28)	369 (39.72)	
Gender				**<0.001**
Male	592 (63.7)	392 (70.0)	200 (54.2)	
Female	337 (36.3)	168 (30.0)	169 (45.8)	
Age (years)				**0.008**
21–28	202 (21.7)	103 (18.4)	99 (26.8)	
29–40	486 (52.3)	303 (54.1)	183 (49.6)	
41–60	241 (26.0)	154 (27.5)	87 (23.6)	
Marriage status				**<0.029**
Single	331 (35.6)	184 (32.9)	147 (39.8)	
Married	598 (64.4)	376 (67.1)	222 (60.2)	
Education level				**<0.029**
High school and below	163 (17.6)	113 (20.2)	50 (13.6)	
Post-secondary	194 (20.8)	122 (21.8)	72 (19.5)	
Undergraduate	427 (46.0)	244 (43.6)	183 (49.6)	
Postgraduate and above	145 (15.6)	81 (14.4)	64 (17.3)	
Exercising status				**<0.001**
Non-exercises	567 (61.0)	316 (56.4)	251 (68.0)	
Exercises	362 (39.0)	244 (43.6)	118 (32.0)	
Smoking status				0.075
No	757 (81.5)	446 (79.6)	311 (84.3)	
Yes	172 (18.5)	114 (20.4)	58 (15.7)	
Drinking status				**0.002**
No	822 (88.5)	481 (85.9)	341 (92.4)	
Yes	107 (11.5)	79 (14.1)	28 (7.6)	
Length of employment (years)				0.474
< 10	571 (61.5)	339 (60.5)	232 (62.9)	
≥ 10	358 (38.5)	221 (39.5)	137 (37.1)	
Weekly working hours (hours)				**<0.001**
−40	93 (10.0)	72 (12.9)	21 (5.7)	
41–48	459 (49.4)	280 (50.0)	179 (48.5)	
49–56	227 (24.4)	117 (20.9)	110 (29.8)	
57–	150 (16.2)	91 (16.3)	59 (16.0)	
Occupational stress				**<0.001**
No	416 (44.8)	286 (51.1)	130 (35.2)	
Yes	513 (55.2)	274 (48.9)	239 (64.8)	
Sleep quality				**<0.001**
Poor-quality	564 (60.7)	268 (47.9)	296 (80.2)	
High-quality	365 (39.3)	292 (52.1)	73 (19.8)	

Bold values indicate statistical significance at *P* < 0.05.

### Associations between weekly work hours, occupational stress, sleep quality, and cervical spine dysfunction

The associations of weekly work hours, occupational stress, and sleep quality with cervical spine dysfunction are shown in Table 2. After adjustment for potential confounders, longer weekly work hours were significantly associated with a higher odds of cervical spine dysfunction compared with working ≤40 h per week. Participants reporting occupational stress had a significantly higher likelihood of cervical spine dysfunction than those without occupational stress. In contrast, good sleep quality was associated with a significantly lower odds of cervical spine dysfunction. Results were based on company-level cluster-robust standard errors.

**Table 2:** Associations of weekly working hours, occupational stress and sleep quality with cervical spine dysfunction.

Variable	*β*	OR (95%CI)^a^	*P* ^a^
Weekly working hours (hours)			
−40		Ref	
41–48	0.78	2.18 (1.30, 3.65)	**0.003**
49–56	0.99	2.70 (1.47, 4.98)	**0.001**
57–	0.74	2.10 (1.25, 3.54)	**0.005**
Occupational stress			
No		Ref	
Yes	0.48	1.62 (1.29, 2.03)	**<0.001**
Sleep quality			
Poor-quality		Ref	
High-quality	−1.40	0.25 (0.19, 0.32)	**<0.001**

^a^Cluster-robust standard errors were computed at the company level. The model was adjusted for age, gender, marriage status, education level, smoking status, drinking status, exercising status and length of employment.

Bold values indicate statistical significance at *P* < 0.05.

Variance inflation factors for all independent variables were below 2, indicating no evidence of significant multicollinearity.

### Associations of weekly work hours, with occupational stress, and sleep quality

Further analyses examined the associations between weekly work hours and occupational stress as well as sleep quality (Table 3). Longer weekly work hours were significantly associated with increased odds of occupational stress and poorer sleep quality after adjustment for covariates. Results were based on company-level cluster-robust standard errors.

**Table 3:** Associations of weekly working hours with occupational stress and sleep quality.

Variable	Occupational stress			Sleep quality		
	*β*	OR (95%CI) ^a^	*P* ^a^	*β*	OR (95%CI) ^a^	*P* ^a^
Weekly working hours (hours)						
−40		Ref			Ref	
41–48	0.50	1.65 (1.21, 2.25)	**0.002**	−0.41	0.66 (0.55, 0.80)	**<0.001**
49–56	0.86	2.35 (1.74, 3.17)	**<0.001**	−0.84	0.43 (0.32, 0.58)	**<0.001**
57–	0.91	2.48 (1.75, 3.52)	**<0.001**	−0.54	0.58 (0.33, 1.03)	0.065

^a^Cluster-robust standard errors were computed at the company level. The model was adjusted for age, gender, marriage status, education level, smoking status, drinking status, exercising status and length of employment.

Bold values indicate statistical significance at *P* < 0.05.

### Mediation analysis

**Figure 1:**
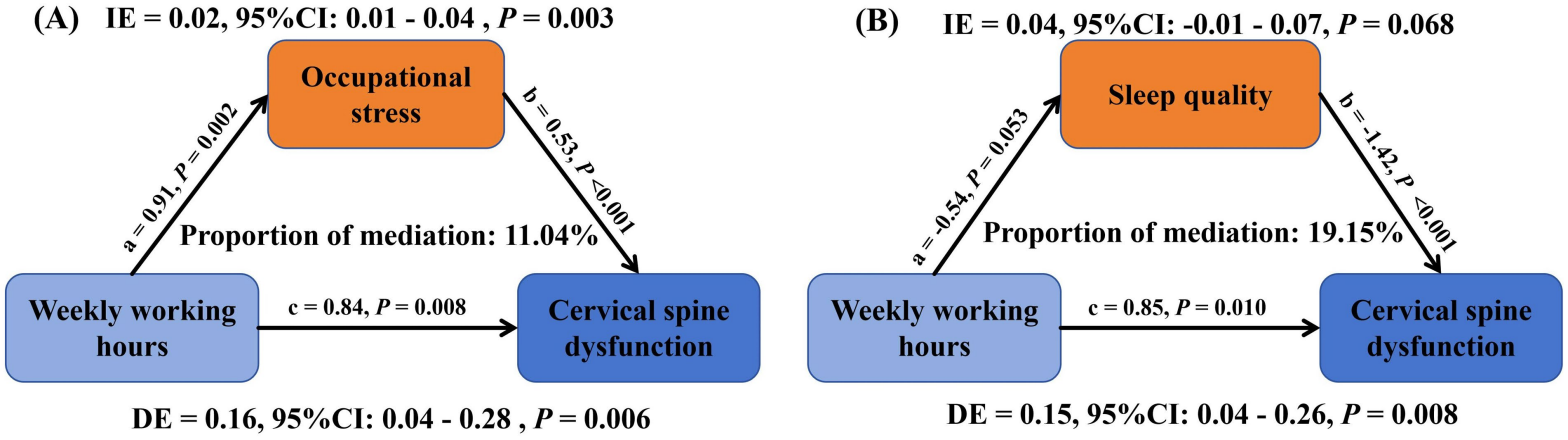
Path diagram for mediated effect analysis in single-mediator models. **(A)** Weekly work hours–occupational stress–cervical spine dysfunction; **(B)** weekly work hours–sleep quality–cervical spine dysfunction. Path coefficients (a-, b-, and c-paths) are shown with corresponding *p* values. IE, indirect effect; DE, direct effect. IE/DE are reported as model-based coefficients (95% CIs) on the same scale as the path estimates.

**Figure 2:**
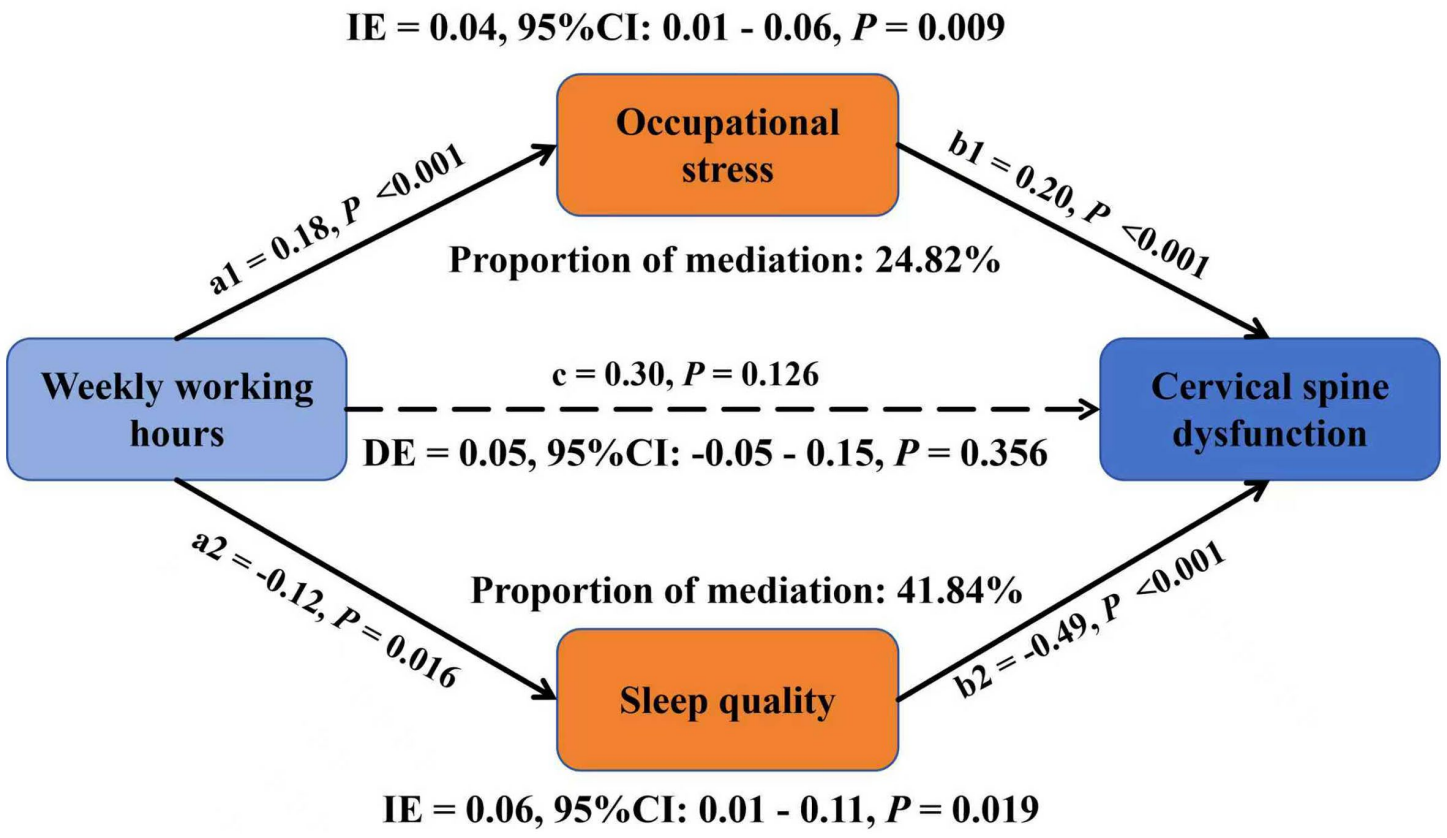
Path diagram for mediated effect analysis in parallel mediator models. Path coefficients (a–, b–, and c-paths) are shown with corresponding *p* values. IE, indirect effect; DE, direct effect. IE/DE are reported as model-based coefficients (95% CIs) on the same scale as the path estimates.

The results of the mediation analyses are presented in Figures 1, 2. In single-mediator models, occupational stress partially mediated the association between weekly work hours and cervical spine dysfunction, whereas the mediating effect of sleep quality was marginal. In the parallel mediation model including both occupational stress and sleep quality, both mediators showed statistically significant indirect effects. After accounting for these indirect pathways, the direct association between weekly work hours and cervical spine dysfunction was no longer statistically significant.

Because the association between weekly work hours and cervical spine dysfunction was non-monotonic across categories, we conducted an additional parallel mediation model specifying weekly work hours as dummy-coded categories (≤40 h/week as reference). The indirect effects and total effects across work-hour groups showed an inverted U-shaped pattern, with the largest total effect observed for 49–56 h/week (Supplementary Table S1). A trend model using orthogonal polynomial coding further supported a significant quadratic component, consistent with nonlinearity (Supplementary Table S2). A sensitivity analysis using continuous mediators (JCQ_rate and PSQI_score) yielded consistent findings, with statistically significant indirect effects and a non-significant direct effect (Supplementary Table S3). A serial mediation model was further examined; however, neither the path from occupational stress to sleep quality nor the corresponding sequential indirect effect was statistically significant, providing no support for a serial mediation pathway (Supplementary Table S4). Additional Spearman correlation analyses showed that PSQI total score, NDI total score, and the JCQ demand–control ratio were positively correlated, with a moderate correlation between PSQI and NDI scores and weaker correlations for the other variable pairs (Supplementary Table S5).

## Discussion

This study examined the association between weekly work hours and cervical spine dysfunction among video display terminal workers and explored the potential roles of occupational stress and sleep quality. Overall, longer weekly work hours were associated with higher odds of cervical spine dysfunction, and mediation analyses were consistent with indirect pathways involving occupational stress and sleep quality.

Consistent with previous studies reporting positive associations between long work hours and work-related musculoskeletal disorders (24, 25), longer weekly work hours were associated with higher odds of cervical spine dysfunction in the present study. Prolonged exposure to video display terminal work is commonly characterized by sustained static loading, constrained neck postures, and repetitive movements, which may contribute to persistent neck muscle tension and increased biomechanical stress on cervical structures and surrounding soft tissues (1, 6, 26). Moreover, extended work hours may reduce opportunities for adequate rest and recovery, thereby potentially exacerbating cumulative musculoskeletal strain and being associated with cervical spine dysfunction (27). From a pathophysiological perspective, prolonged VDT work may promote neck disability through sustained forward-head posture and low-level static contraction of the cervical extensor and shoulder girdle muscles, which can impair local microcirculation, accelerate muscle fatigue, and increase the accumulation of pain-related metabolites. Over time, these responses may contribute to pain, reduced range of motion, and functional limitation in the cervical region, thereby increasing NDI-defined neck disability. Notably, the association remained significant after adjustment for a wide range of demographic, lifestyle, and occupational factors, suggesting that long work hours may represent an independent correlate of cervical spine dysfunction. Awkward neck posture is widely regarded as a key biomechanical risk factor for cervical musculoskeletal problems in VDT work. Because posture- and workstation-related exposures were not measured in this study, residual confounding cannot be excluded, and these unmeasured biomechanical factors may partly contribute to the observed associations.

Occupational stress appeared to be an important psychosocial factor potentially involved in the association between long work hours and cervical spine dysfunction in this study. This observation is in line with previous evidence showing that psychosocial work stressors may contribute to musculoskeletal health outcomes (4, 5, 28, 29). From a mechanistic perspective, sustained occupational stress has been linked to prolonged muscle tension, heightened pain sensitivity, and dysregulation of neuroendocrine responses, all of which may exacerbate the burden on cervical muscles and surrounding soft tissues (30, 31). In addition, occupational stress may affect pain perception, coping strategies, and health-related behaviors, thereby potentially amplifying the adverse effects of long work hours on cervical spine function. Consistent with previous studies examining the relationship between occupational stress and work-related musculoskeletal disorders, our findings underscore the importance of psychosocial factors in cervical spine dysfunction.

Sleep quality also appeared to be an important behavioral correlate of cervical spine dysfunction in relation to long work hours. This pattern is consistent with previous studies indicating that sleep-related factors are closely intertwined with psychosocial stress and may exert stronger effects when examined within integrated analytical frameworks (32, 33). From both biological and behavioral perspectives, adequate sleep is essential for musculoskeletal recovery and functional restoration. Poor sleep quality may impair muscle repair processes, disrupt inflammatory regulation, and alter pain perception, thereby potentially contributing to the accumulation of musculoskeletal fatigue and the development of chronic pain (34, 35). Long work hours are often accompanied by reduced sleep duration and compromised sleep quality, which may weaken the body’s restorative capacity and increase vulnerability of cervical structures and surrounding soft tissues to injury. Consistent with previous research highlighting the role of sleep quality in work-related musculoskeletal disorders, our findings further emphasize the importance of sleep health in the prevention of cervical spine dysfunction (7, 32, 36). These supplementary correlation results further support the interrelationships among occupational stress, sleep quality, and neck disability observed in the present study.

In the parallel mediation model, the direct effect of weekly work hours on cervical spine dysfunction was no longer statistically significant after simultaneously accounting for occupational stress and sleep quality, suggesting that the observed association may be largely consistent with indirect pathways. Such coexisting psychosocial and behavioral pathways have been increasingly emphasized in contemporary occupational health research (37). Occupational stress may increase vulnerability to musculoskeletal strain by affecting neuroendocrine regulation and muscle tension (38), while impaired sleep quality may compromise recovery processes and exacerbate cumulative musculoskeletal damage (34, 35). In addition, the non-monotonic pattern across work-hour categories may reflect heterogeneity in job tasks, adaptation, or selection effects (e.g., healthier workers remaining in very long-hour positions), and warrants confirmation in longitudinal studies. The main findings were robust in sensitivity analyses using continuous JCQ and PSQI measures, reducing concern that dichotomization materially influenced the conclusions.

The findings have important public health and occupational health implications. While interventions often emphasize working-time reduction or ergonomic modification, our results suggest that addressing working hours alone may be insufficient to reduce the odds of cervical spine dysfunction. Accordingly, prevention strategies could be considered that integrate work organization measures to mitigate psychosocial stress, stress-management programs, and sleep health education and behavioral interventions (39, 40). By simultaneously addressing organizational, psychological, and behavioral factors, more effective strategies for reducing the burden of cervical spine dysfunction among VDT workers may be achievable.

Several strengths of this study should be acknowledged. First, this study systematically examined the potential mediating roles of occupational stress and sleep quality in the association between weekly work hours and cervical spine dysfunction, and employed a parallel mediation model to characterize the combined effects of psychosocial and behavioral factors. Second, a wide range of demographic, lifestyle, and occupational variables were adjusted for, enhancing the robustness of the findings. In addition, the focus on VDT workers provides occupation-specific evidence relevant to cervical spine health in this population.

Nevertheless, several limitations should be considered. The cross-sectional design precludes definitive conclusions regarding causal relationships, and causal inferences should therefore be made with caution. Furthermore, occupational stress, sleep quality, and cervical spine dysfunction were assessed using self-reported questionnaires, which may be subject to recall or reporting bias. Because participants were recruited from a limited number of companies, the generalizability of the findings may be constrained, although the inclusion of diverse VDT workplaces may partially mitigate this limitation. Finally, although multiple confounders were adjusted for, residual confounding by unmeasured factors (e.g., awkward neck posture and workstation ergonomics, as well as mental health comorbidities) cannot be entirely ruled out, which may bias both the association estimates and the indirect effect estimates. Future longitudinal studies incorporating objective measurements are warranted to confirm and extend these findings.

## Conclusion

This study found that longer weekly work hours were significantly associated with higher odds of cervical spine dysfunction among video display terminal workers. Mediation analyses suggested that occupational stress and sleep quality may represent potential indirect pathways linking weekly work hours with cervical spine dysfunction. The observed association may not be explained by biomechanical workload alone and may also be partly related to psychosocial stress and sleep quality. Prevention strategies should integrate working-time management with ergonomic interventions targeting neck posture, alongside occupational stress reduction and sleep health promotion.

## Data Availability

The raw data supporting the conclusions of this article will be made available by the authors, without undue reservation.
