# Supplementary material for: Weekly work hours and cervical spine dysfunction among video display terminal workers: mediating roles of occupational stress and sleep quality
Source: Front Public Health. 2026 Jun 1;14:1798795. doi: 10.3389/fpubh.2026.1798795 (PMC13265529; doi:10.3389/fpubh.2026.1798795)
Supplement: Supplementary file 1 [file Supplementary_file_1.docx]

Supplementary Tables

S1 Results of the Parallel Mediation Model (Classification)

| Path/effect | Label | Estimate | Std. Err | *P* | 95% CI | Std. All |
| --- | --- | --- | --- | --- | --- | --- |
| **Independent variable → Mediator (path a)** | | |  |  |  |  |
| 41-48 h → JCQ | a1_2 | 0.308 | 0.145 | **0.034** | (0.023, 0.593) | 0.145 |
| 49-56 h → JCQ | a1_3 | 0.528 | 0.161 | **0.001** | (0.215, 0.841) | 0.213 |
| 57- h → JCQ | a1_4 | 0.561 | 0.173 | **0.001** | (0.221, 0.900) | 0.194 |
| 41-48 h → PSQI | a2_2 | -0.255 | 0.149 | 0.087 | (-0.547, 0.037) | -0.122 |
| 49-56 h → PSQI | a2_3 | -0.516 | 0.165 | **0.002** | (-0.838, -0.193) | -0.212 |
| 57- h → PSQI | a2_4 | -0.337 | 0.177 | 0.058 | (-0.684, 0.011) | -0.119 |
| **Mediator → Dependent (path b)** | |  |  |  |  |  |
| JCQ → NDI | b1 | 0.193 | 0.053 | **<0.001** | (0.089, 0.297) | 0.192 |
| PSQI → NDI | b2 | -0.483 | 0.046 | **<0.001** | (-0.573, -0.394) | -0.472 |
| **Indirect effect** |  |  |  |  |  |  |
| 41-48 h vs -40 h | Indirect 2 vs 1 | 0.183 | 0.078 | **0.019** | (0.030, 0.335) | 0.085 |
| 49-56 h vs -40 h | Indirect 3 vs 1 | 0.351 | 0.091 | **<0.001** | (0.173, 0.530) | 0.141 |
| 57- hvs -40 h | Indirect 4 vs 1 | 0.271 | 0.096 | **0.005** | (0.083, 0.459) | 0.093 |
| **Total effect (direct + indirect)** | |  |  |  |  |  |
| 41-48 h vs -40 h | total_2_vs_1 | 0.533 | 0.164 | **0.001** | (0.212, 0.854) | 0.249 |
| 49-56 h vs -40 h | total_3_vs_1 | 0.756 | 0.175 | **<0.001** | (0.414, 1.099) | 0.304 |
| 57- h vs -40 h | total_4_vs_1 | 0.566 | 0.189 | **0.003** | (0.195, 0.936) | 0.195 |

S2 Results of parallel mediation model effects based on orthogonal polynomial coding

| Path/effect | Label | Estimate | Std. Err | *P* | 95% CI | Std. All |
| --- | --- | --- | --- | --- | --- | --- |
| **Independent variable → Mediator (path a)** | | |  |  |  |  |
| work hours (linear) → JCQ | a1_l | 0.097 | 0.025 | <0.001 | (0.047, 0.147) | 0.16 |
| work hours (quadratic) → JCQ | a1_q | -0.068 | 0.05 | 0.178 | (-0.166, 0.031) | -0.056 |
| work hours (linear) → PSQI | a2_l | -0.073 | 0.026 | 0.005 | (-0.124, -0.021) | -0.122 |
| work hours (quadratic) → PSQI | a2_q | 0.103 | 0.051 | 0.045 | (0.002, 0.203) | 0.086 |
| **Mediator → Dependent (path b)** | |  |  |  |  |  |
| JCQ → NDI | b1 | 0.193 | 0.053 | <0.001 | (0.089, 0.298) | 0.192 |
| PSQI → NDI | b2 | -0.483 | 0.046 | <0.001 | (-0.572, -0.394) | -0.471 |
| **Indirect effect** |  |  |  |  |  |  |
| indirect effect of linear | indirect_l | 0.054 | 0.015 | <0.001 | (0.025, 0.083) | 0.088 |
| indirect effect of quadratic | indirect_q | -0.063 | 0.027 | 0.019 | (-0.115, -0.010) | -0.051 |
| **Effect decomposition** | |  |  |  |  |  |
| total indirect effect | total_ind | -0.009 | 0.027 | 0.741 | (-0.061, 0.043) | 0.037 |
| total direct effect | total_d | -0.073 | 0.05 | 0.141 | (-0.171, 0.024) | -0.023 |
| total effect | total_eff | -0.082 | 0.052 | 0.111 | (-0.183, 0.019) | 0.014 |

S3 Results of Parallel Mediation Model (Continuous Mediating Variables)

| Path/effect | Label | Estimate | Std. Err | *P* | 95% CI | Std. All |
| --- | --- | --- | --- | --- | --- | --- |
| **Independent variable → Mediator (path a)** | | |  |  |  |  |
| work hours → JCQ_rate | a1 | 0.048 | 0.009 | <0.001 | (0.030, 0.067) | 0.166 |
| work hours → PSQI_score | a2 | 0.400 | 0.124 | 0.001 | (0.156, 0.643) | 0.112 |
| **Mediator → Dependent (path b)** | | |  |  |  |  |
| JCQ_rate → NDI | b1 | 0.803 | 0.166 | <0.001 | (0.477, 1.129) | 0.193 |
| PSQI_score → NDI | b2 | 0.165 | 0.010 | <0.001 | (0.146, 0.185) | 0.493 |
| **Direct effect (path c)** | |  |  |  |  |  |
| work hours → NDI | c | 0.037 | 0.048 | 0.444 | (-0.057, 0.130) | 0.03 |
| **Indirect effect** |  |  |  |  |  |  |
| through JCQ_rate | indirect1 | 0.039 | 0.011 | <0.001 | (0.017, 0.060) | 0.032 |
| through PSQI_score | indirect2 | 0.066 | 0.021 | 0.002 | (0.025, 0.107) | 0.055 |
| total indirect effect | total_ind | 0.105 | 0.025 | <0.001 | (0.056, 0.154) | 0.087 |
| **Total effect** | total_eff | 0.141 | 0.052 | 0.006 | (0.040, 0.242) | 0.118 |

S4 Results of the Chain Mediation Model

| Path/effect | Label | Estimate | Std. Err | *P* | 95% CI | Std. All |
| --- | --- | --- | --- | --- | --- | --- |
| **Path a** |  |  |  |  |  |  |
| work hours → JCQ | a | 0.17 | 0.05 | **0.001** | (0.072, 0.268) | 0.139 |
| **Path b** |  |  |  |  |  |  |
| JCQ → PSQI | b | -0.07 | 0.055 | 0.205 | (-0.178, 0.038) | -0.071 |
| **Path c** |  |  |  |  |  |  |
| PSQI → NDI | c | -0.462 | 0.047 | **<0.001** | (-0.554, -0.370) | -0.457 |
| **Else direct path** |  |  |  |  |  |  |
| JCQ → NDI | b1 | 0.151 | 0.053 | **0.004** | (0.048, 0.254) | 0.152 |
| work hours → PSQI | a2 | -0.075 | 0.053 | 0.157 | (-0.178, 0.029) | -0.062 |
| **Direct effect** |  |  |  |  |  |  |
| work hours → NDI | d | 0.049 | 0.052 | 0.344 | (-0.053, 0.152) | 0.041 |
| **Indirect effect** |  |  |  |  |  |  |
| Through JCQ (separately) | indirect1 | 0.026 | 0.012 | **0.027** | (0.003, 0.048) | 0.021 |
| JCQ → PSQI (chain) | indirect2 | 0.005 | 0.005 | 0.231 | (-0.003, 0.014) | 0.005 |
| total indirect effect | total_indirect | 0.031 | 0.013 | **0.016** | (0.006, 0.056) | 0.026 |
| **Total effect** | total_effect | 0.08 | 0.052 | 0.12 | (-0.021, 0.182) | 0.066 |

S5 Spearman correlation analysis among PSQI total score, NDI total score, and JCQ demand–control ratio

| Variables | Spearman’s *ρ* | 95% CI | *P* |
| --- | --- | --- | --- |
| PSQI total score vs. NDI total score | 0.49 | (0.44, 0.54) | < 0.001 |
| JCQ demand–control ratio vs. NDI total score | 0.23 | (0.17, 0.29) | < 0.001 |
| PSQI total score vs. JCQ demand–control ratio | 0.15 | (0.09, 0.22) | < 0.001 |

Note: Correlations were estimated using Spearman’s rank correlation coefficients.
